# Needs of Rural Caregivers of Patients With Cancer: A Scoping Review of Evidence From North America, Europe, and Australia

**DOI:** 10.1002/pon.70615

**Published:** 2026-10-07

**Authors:** Jeanne M. Ward, Christa Torrisi, Nicole Werner, Victoria Champion

**Affiliations:** ^1^ Indiana University School of Nursing Indianapolis Indiana USA; ^2^ Department of Anesthesiology Vanderbilt University Medical Center Nashville Tennessee USA

**Keywords:** caregiver burden, caregivers, caregiver stress, mental health, psycho‐oncology, rural health

## Abstract

**Introduction:**

Informal caregivers (caregivers) in rural areas experience many stressors affecting their mental and physical health. This review will synthesize the current needs of cancer caregivers in rural areas to inform future research and interventions.

**Methods:**

Five databases (PsycInfo, CINAHL, EMBASE, PubMed, and Family & Society Studies Worldwide) were queried to identify studies examining mental health and quality‐of‐life outcomes in rural caregivers of cancer patients worldwide, using the Arksey and O'Malley principled analytic framework. A priori criteria included rural caregivers of adult cancer patients in peer‐reviewed studies from 2005 to 2025.

**Results:**

Database searches yielded 337 records. After title and abstract screening, 134 articles were reviewed, and of these, 35 met a priori criteria. The most frequent problems identified among rural cancer caregivers were mental health issues (depression and anxiety), loneliness or isolation, fatigue and physical issues, provider issues, and financial issues, especially related to travel, balancing duties, and cultural differences between rural populations and urban providers. Most articles were from outside the U.S.; however, results from rural caregivers in the U.S. described needs reflected in literature from other countries.

**Conclusions:**

Given increased utilization of caregivers in the U.S. and the rural burden of cancer, it is crucial to identify caregivers' unmet needs. Home‐based cancer care is increasing, necessitating additional studies to determine needs and develop effective interventions for rural caregivers. Rurally competent care from healthcare providers who interface with caregivers of their cancer patients is vital, as caregivers are well‐positioned to partner with providers to improve the well‐being of those patients.

## 1 Introduction

U.S. cancer incidence is rising, with two million diagnoses expected in 2025 [1]. Due to healthcare system changes, and a higher proportion of older adults in the population, cancer patient caregiving is increasingly shifting to informal caregivers (caregivers), unpaid family members or friends assisting the patient in the home [2]. For purposes of this review, the term “caregivers” refers to informal caregivers who provide at least 5 hours per week of unpaid care to a cancer patient and who may or may not reside with the person who has cancer. In the U.S, approximately four million people serve as cancer patient caregivers [3]. Caregivers directly affect cancer patients' outcomes, especially in mental health and side effects [4]. However, previous research has shown that after assuming the role of cancer patient caregiver, caregivers frequently experience a decline in their own quality of life, experiencing physical and psychological distress, often exceeding the distress of the cancer patient [5].

Rural caregivers face extra challenges, and rural areas have a higher burden of cancer [6]. A higher prevalence of individuals with cancer is in rural compared to urban areas [7]. Rural life presents unique challenges, such as increased travel distances and other disparities, such as income and barriers to healthcare access, delaying cancer screenings and diagnoses, often worsening outcomes [6].

Cancer patient caregivers' general needs have been identified, including skills training, provider communications, healthcare system navigation, and health and well‐being [8]. Cancer caregivers have more mental health needs than the general population [9], and among the highest caregiver levels of psychological and physical burden [10]. This burden is from the intense patient caring load, requiring reacting to multiple needs, from activities of daily living, such as feeding, dressing, and toileting a previously independent individual, to nursing tasks, such as medication administration and injections, ostomy and catheter care, and tube feedings [3].

Although general needs have been identified, there has not been a recent thorough investigation of the current needs of rural caregivers, with the most recent review published in 2004 [11], and the only other recent reviews about cancer caregivers being nonspecific to rural needs, and covering specific caregiver types, such as pancreatic [12] and adolescent/young adult cancers [13], or specific aspects of cancer caregivers, such as depression, quality of life, social support, and educational programs [14, 15, 16, 17]. Given cancer control and treatment and technology, the cancer care landscape has changed over the last 2 decades, with care increasingly provided on a digital and outpatient basis [18]. Rural caregiver needs differ from those of urban caregivers due to heightened geographic challenges, creating travel barriers and social isolation. Furthermore, rural individuals exhibit distinct health‐seeking behaviors and cultural factors affecting health outcomes, leading to a reluctance to seek healthcare [6]. Alongside these disparities, rural cancer caregivers are disproportionately highly represented [19]. Therefore, a scoping review will synthesize current literature and inform future research to improve services and develop innovative solutions to address challenges of rural caregiving [20].

The objective of this study was to conduct a scoping review of recent literature on rural cancer patient caregivers' needs by synthesizing recent evidence from North America, Europe, and Australia. By focusing on regions with broadly comparable healthcare delivery and caregiving contexts, this review will guide future U.S‐focused research investigating rural cancer caregivers' needs. This will provide healthcare providers with relevant information to better serve rural cancer patients and their caregivers, and an intervention development basis.

## 2 Methods

A scoping review's goal is to describe trends and identify gaps in a broad research area, exploring broader topics than a systematic review, enriching topic understanding. An a priori protocol for the scoping review was not registered in a public repository such as the Open Science Framework. However, it was conducted with the principled analytic framework by Arksey and O'Malley (2005), comprised of the following five steps: identifying the research question; identifying relevant studies; selecting studies; charting data; and collecting, summarizing, and reporting the results [21]. The review methods, including eligibility criteria, search strategy, and approach to data extraction, were established before the study selection.

Five databases were used for article searches: PsycINFO, CINAHL, EMBASE, PubMed, and Family & Society Studies Worldwide. The articles were restricted to 2005–2025 to ensure relevance to the recent cancer care landscape. In the last 20 years, cancer treatment and care delivery have undergone substantial changes, shifting toward outpatient and home‐based care, increased use of oral therapies, expanded reliance on informal caregivers, and rapid growth of digital and telehealth services [2, 4]. Studies conducted prior to 2005 may not reflect current caregiving responsibilities, healthcare access patterns, or systems‐level factors influencing rural cancer caregiving, and scoping review best practices recommend using current, relevant evidence to identify gaps [22]. As this scoping review was intended to describe the nature of the current literature discussing aspects of problems affecting rural caregivers of cancer patients, the study eligibility criteria were: (1) population included informal caregivers of adult cancer patients; (2) informal caregiver needs and problems were assessed; (3) sample was partially or fully assessed as rural; (4) peer‐reviewed study from an academic journal; and (5) full‐text available in English. Exclusion criteria included reviews, pediatric studies, intervention evaluations, studies not mentioning rurality, and studies assessing bereavement surrounding informal caregiving. Studies primarily sampling bereaved or pediatric caregivers were excluded. Furthermore, studies from countries outside North America, Europe, and Australia were excluded to focus the review on regions with the most comparable healthcare delivery, access challenges, and informal caregiving contexts, given that the review aimed to inform future research on rural U.S. caregivers. Only full‐text articles in English were included to ensure accurate interpretation and consistency in data extraction by the review team.

The keywords, developed in consultation with a university librarian and study reviewers, included the term “caregiver” and other groups of search terms using the Boolean operator “and” to identify studies including caregiver needs or problems and rurality. These terms were used to encompass caregiving headings, utilizing MeSH terms and headings for parsimony. One author executed the search strategy after agreement with the other article reviewer. The initial search was conducted in February 2025, and a follow‐up was conducted in September 2025 (Table S1).

Duplicate citations were removed, and article titles and abstracts were reviewed to identify articles meeting full‐text review criteria. Next, we reviewed full‐text versions using the same exclusion categories. For both steps —article title, abstract review, and full‐text review —two authors (J.W., C.T.) independently screened the studies separately to identify differences and cross‐validate extracted information. Differences at the abstract or full‐text review stage were discussed and resolved through discussion and review of the eligibility criteria to achieve consensus on the included studies. To reduce potential bias in the search strategy, previously excluded review articles and the utilized articles' reference lists were reviewed to identify additional studies meeting inclusion criteria. Finally, all studies were searched for citations on Google Scholar.

For each article meeting criteria, information was extracted, which included: the country, year of publication, authors, sample demographics, study design, patient cancer type, rural description, and major findings such as needs and problems, and specific rural contextual factors. Extracted data were charted and synthesized using the Arksey & O'Malley principled analytical framework [21]. This framework structured the extraction across caregiving problems, needs, and context to identify caregivers' unmet needs. Rural contextual factors were defined as structural, healthcare system, economic, and cultural conditions associated with rurality that shape caregiver demands and challenges.

## 3 Results

After duplicate screening, 337 abstracts and titles were reviewed, and 134 articles received a full‐text review. Additionally, reference list reviews yielded four articles, Google Scholar yielded 19 articles, and a second search of all databases in September 2025 yielded two additional articles. Finally, 35 articles met the criteria for extraction. Figure 1 describes the process of study selection. In the results, we identify commonalities and differences among the characteristics of the identified studies. We present a list of all studies (Table 1) with identified major themes, highlighting the specific problems and needs facing rural cancer caregivers.

**FIGURE 1:**
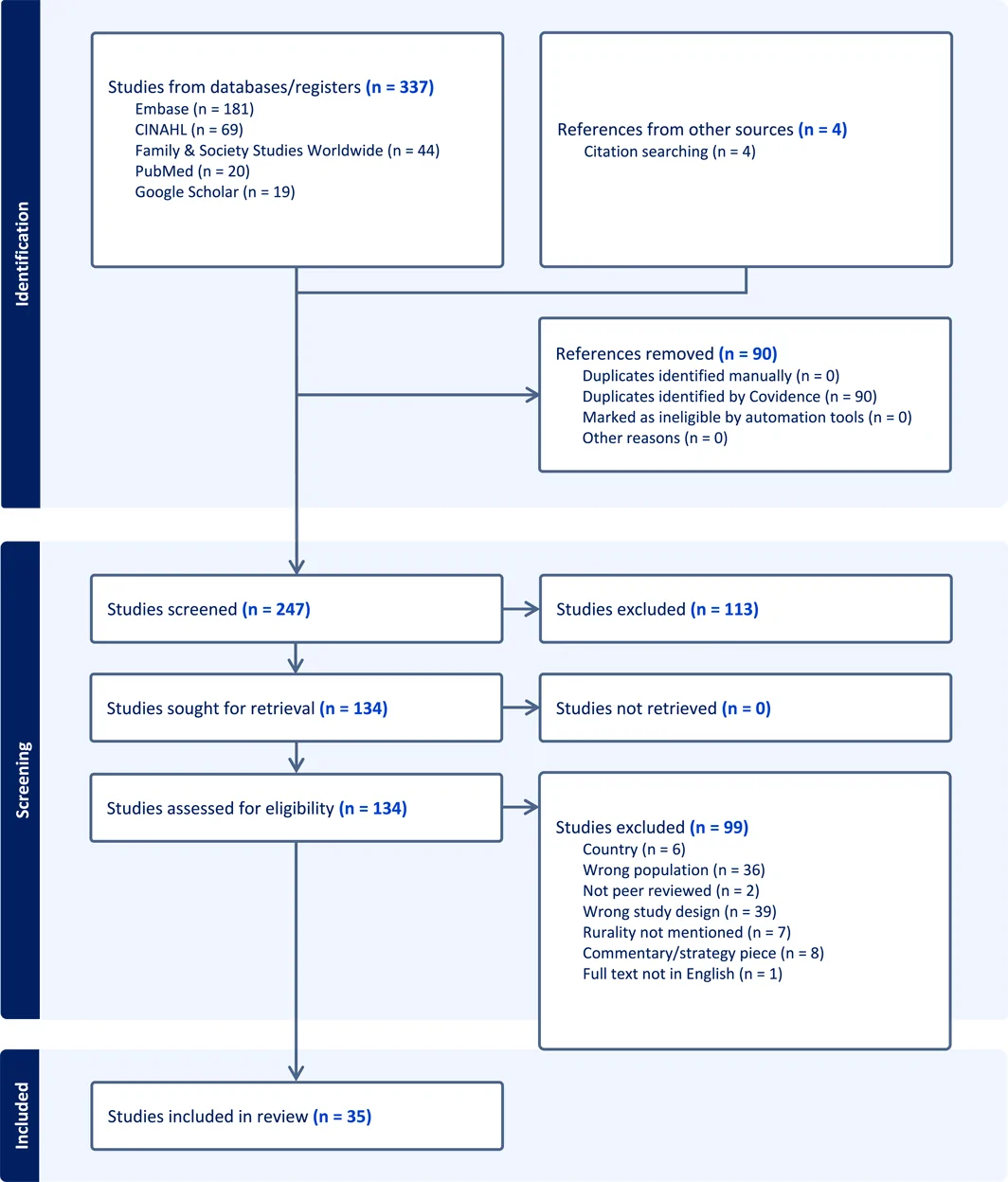
Needs of rural cancer caregivers.

**TABLE 1:** Table of studies.

Study author, year	Location	Purpose	Design	Sample size of ICs	Sample demographics	Rurality notes	Cancer type/stage (%)	Principal findings (identified problems and needs)	Rural contextual factors
Barnes et al. 2023 [23]	Australia	Explore symptoms and psychological distress of rural cancer patients and caregivers, and barriers to accessing psychosocial oncology support.	Mixed methods	34	Combined caregivers and patients: Age mean = 60 years 81% female	Affiliated with regional/rural treatment centers; rurality confirmed by postcode (Australian statistical geography standard).	40% breast, 11% prostate 11% cervical/uterine/ovarian 8% colorectal 8% blood cancers 4% melanomas 3% lung 2% esophageal 13% less common Stage not indicated	*Problems*: Mental distress (depression/stress) of caregivers is high; confidentiality concerns frequent; caregivers ineligible/low priority for services; physical exhaustion, loneliness.	Rural access/availability low disconnected with urban providers; financial strain due to time off work; stoicism and self‐reliance norms inhibit help‐seeking
Beddard et al. 2025 [24]	United Kingdom	Explore perspectives of bowel cancer patients and caregivers in wales.	Mixed methods, survey and interviews, focus groups	20 (survey), 5 (interviews/focus groups)	Survey: 30% age 31–40 years, 90% female, 100% white	Differentiated by counties in wales, primarily rural, definition unclear.	Unspecified. Survey: 30% stage 4, 15% stage 3, 5% stage 2, 10% stage 1	*Problems*: challenges balancing full‐time work and parenting responsibilities. *Needs*: Mental health support.	Lower care quality and continuity of care; difficulties accessing support in rural counties; limited specialists and survivorship support; travel. Logistics
Brazil et al. 2013 [25]	Canada	Measure caregiver health status, burden, social supports, and use of formal care.	Cross‐sectional survey	100	Age mean = 56.5 years(SD = 10.4) 88.6% female,	44% rural, defined as living outside designated urban centers, communities with fewer than 30,000 residents.	Unspecified	*Problems*: Frequently caregivers needed to relocate. *Needs*: Respite services for rural caregivers.	Long travel times, lower access to specialists, delayed support from overextended providers
Breen et al. 2024 [26]	Australia	Identify the specific unmet psychosocial needs of pleural mesothelioma patients and caregivers.	Mixed methods, survey and interviews	25	Age median = 66 years (range 41–79)	20% rural/regional, definition not described.	Mesothelioma.	*Needs*: Tailored information resources; social and peer support; regular check‐ins from providers; help with managing stress, anxiety, and hopelessness.	Fragmented rural services, lack of access to psychological services
Cormican et al. 2025 [27]	Ireland, Australia, UK, USA, India)	Investigate psychosocial vulnerability among caregivers of chronic blood cancer patients	Cross‐sectional survey	64	50% ages 61–80, 34% ages 41–60 75% female	73% rural, definition not described.	Chronic hematological cancers.	Problems: Younger caregivers had more stress and strain; more loneliness among caregivers of patients currently receiving treatment; financial and employment difficulties.	High travel burden; employment strain
Duggleby et al. 2014 [28]	Canada	Determine factors associated with hope for rural women caregivers of individuals with advanced cancer, looking at demographics, self‐efficacy, grief, and caregiver physical/mental health.	Cross‐sectional survey	122	Age mean = 59 years (11.5), mean education years = 13 (2.4), 76.2% married, 82.8% caucasian, 100% female	All rural, determined by postal code community of < 10,000 (Saskatchewan) or does not live in large population centers (Alberta)	Advanced cancer 13.1% other 13.1% colorectal 13.1% lymphoma 10.7% lung 9.8% prostate 7.4% urological 4.9% nasopharyngeal 3.3% breast	*Problems*: Caregiver mental and physical health below population norms *Needs*: Self‐efficacy and hope.	Limited care access; isolation from care centers; scarce counseling access; self‐reliance
Duimering et al. (2020) [29]	Canada	Investigate quality of life of caregivers of patients in a palliative radiotherapy program.	Cross‐sectional survey	201	Age mean = 58.4 years (SD = 14) 60.8% female	31% rural, definition not described	25% lung 19% prostate 19% breast 6% colorectal 5% renal 5% bone 3% sarcoma 3% lymphoma	*Problems*: Employment and living situations affect distress. *Needs*: Support around childcare	Distance and access barriers
Gaugler et al. 2005 [30]	United States, Kentucky and Maryland	Identify factors accounting for multiple dimensions of caregiver subjective stress.	Cross‐sectional survey	130 from Kentucky, 103 from Maryland	In KY group Age mean = 53.85 years (12.79) 70% female 79.2% married 42.3% employed	Included rural areas of Kentucky, but exact percentages not assessed. Maryland participants considered urban.	In KY group: 20.8% colorectal 13.1% pancreatic/liver 12.3% gynecological 8.5% lung (vs. 25.2% in MD) 10% bone or leukemia 8.2% prostate or bladder 6.9% brain 3.1% skin 6.9% breast 2.3% other	*Problems*: Longer hours care, more palliative care, and mood issues in KY group; lack of socioemotional support; role captivity and overload; younger caregivers had more fatigue and physical exhaustion than older caregivers; more caregiver stress if shorter treatment period and if more activities of daily living *Needs*: Mastery of care skills.	Cultural differences among rural caregivers, lower rural education, employment, and income levels,
George et al., 2022 [31]	Australia	Comprehend the effects of rurality on the experience of cancer care in older patients and the impacts of combined rurality and comorbidity for those supporting or providing clinical care to those patients.	Qualitative, focus groups	16	Age mean = 59 years (SD = 11.5) 50% female 76% married 66% caring for spouse, 82.8% white	All rural via two regional locations over 300 km from nearest metropolitan center.	All patients were 65 years or older with solid tumor cancer at any stage receiving treatment. Included breast, bowel, lung, prostate and other cancers.	*Problems*: caregivers may downplay own health or set aside personal issues; lack of close access to staff.	Difficulties with travel led to financial hardship; social and informational isolation; limited local access to treatment and availability.
Goodwin et al. 2022 [32]	Australia	Examine quality of life, depression, anxiety, and stress in caregivers of cancer pts in regional/remote areas versus population norms. Explore drivers of poorer outcomes between remote caregivers, looking at demographics, patient outcomes, and caregiver burden and health.	Cross‐sectional survey	241	Age mean = 62.1 years (12.6). 62.1% female, usually spouse	All regional/rural, defined as getting treatment > 50 km from home. 52.3% inner regional, 42.7% outer regional settings.	20.6% breast 15.2% skin 10.8% prostate 12.1% head/neck 9.4% gynecological 7.2% lung 5.8% non‐hodgkin lymphoma 5.8% brain 10.8% other	*Problems*: Lower quality of life and more depression in caregivers versus general population; higher caregiver burden associated with poorer caregiver mental and physical health, depression, anxiety, and stress.	Social isolation, logistical travel challenges, reduced healthcare access and respite; financial burden increased
Grimison et al. 2013 [33]	Australia	Identify concerns from caregivers about cancer care.	Qualitative, focus groups and interviews	14	Age mean = 68 years 92.9% female	All rural caregivers of patients in Accessibility/Remoteness index of Australia (ARIA) codes 3–5.	Cancer diagnosis last 2 years, 14.2% breast cancer 35.7% colorectal cancer 7.1% lung cancer 7.1% melanoma, half metastatic cancers.	*Problems*: Lack of access to healthcare professionals and services; poorly coordinated services; caregivers felt staff did not understand difficulties related to distance.	Financial hardship due to travel; geographic isolation; limited access to specialists and treatment‐poor after‐hours coverage; social isolation and limited local caregiver support
Gunn et al., 2022 [34]	Australia	Explore the experiences of caregivers for cancer patients in rural areas, the impact of cancer caring on their wellbeing.	Qualitative, interviews	18	Age mean = 66 years 66.7% female	ARIA codes 2–4: 5.6% inner regional, 55.6% outer regional, 38.9% remote	22.2% immune system 22.2% bowel 11% breast 5.5% lung 5.5% brain 5.5% cervical 5.5% prostate 5.5% esophageal 5.5% gallbladder 5.5% unknown 22.2% late stage/palliative	*Problems*: Financial challenges; frustration with healthcare; loss of identity; sadness/grief; isolation; difficulties balancing practical tasks;	Travel challenges and financial strain; specialists and schedules don't understand rural distances; provider issues‐ long waits and rural provider turnover; reduced access to psychological services; gendered rural norms about caregiving
Harrison et al. 2021 [35]	Australia	Explore the role caregivers play in providing clinical care for loved ones with cancer and their views regarding the support they need to contribute to healthcare partnerships.	Qualitative, focus groups and interviews	20	70% female, 50% from culturally and linguistically diverse (CALD) backgrounds	25% of participants from remote or rural areas, definition not described.	Range of cancers, including 25% pediatric neuroblastoma.	*Problems*: Mental distress; burden of clinical care; difficulties with employment and family responsibilities; frequently changing providers; rapid upskilling needed.	Travel & financial challenges; lack of support in rural areas; increased logistical complexity and clinical responsibility; need to leave employment; reduced access to psychosocial and specialist services
Itty et al. 2014 [36]	Southwest United States	Identify and categorize illness beliefs and barriers to symptom management faced by American indian cancer patients/survivors and family members, and to better understand how these constructs impact the cancer experience.	Qualitative, focus groups	126	70.6% female	64.3% urban, 35.7% rural participants. Rural defined as less than 1000 people per square mile.	Cancer types unspecified.	*Problems*: Communication and cultural barriers (e.g., discussions about death, western medicine); structural, physical, and support barriers; financial and job stress; difficulties with childcare arrangements, care for home, don't want to overburden family; care skill lack; loneliness and isolation.	Distinct rural beliefs/culture clashes & limited translation services; transportation challenges given long distances to care; long distances to care; limited support groups & services, social isolation due to reluctance discussing illness
Johnson et al. 2020 [37]	Australia	Explore the characteristics of rural general practice which exemplify optimal end‐of‐life (EOL) care from the perspective of people diagnosed with cancer, their informal carers and general practitioners.	Qualitative, interviews	3 participating in a support group/services, 6 cancer patients, 4 practitioners	Age mean = 59.7 years 100% female	Rural/regional Australia, definition not described.	Advanced cancer but otherwise not assessed.	*Needs*: Trusting relationship with practitioner; more information on symptom and pain management; ideal to have general practitioner view caregiver concerns separately.	Geographic distance and isolation, limited specialist access; poor information transfer/delays among providers; limited healthcare professionals; small community complexities
Johnston et al. 2024a [38]	Australia	Understand rural caregiver experiences seeking support for own health/wellbeing, identify what was or wasn't helpful.	Qualitative, interviews	20	Age median = 58 years 50% female	50% inner regional and 50% outer regional using Australian statistical geographic standards	25% head & neck 20% gynecological 15% breast 15% skin 10% gastrointestinal 5% blood 5% brain 5% lymphoma	*Problems*: High emotional burden; social isolation; unsure of where to get support; balancing daily responsibilities *Needs*: Provider acknowledgment.	Feel like providers do not understand rural needs; access issues; long waits; travel distance and costs; social isolation during travel when relocating temporarily. “Grin & bear it” rural attitude internalizes issues
Johnston et al. 2024b [39]	Australia	Explore how caring for someone with cancer affected rural caregivers' health behaviors. Changes in health behaviors and socioecological factors underlying these.	Qualitative, interviews	20	Age median = 58 years 50% female	50% inner regional and 50% outer regional using Australian statistical geographic standards	25% head & neck 20% gynecological 15% breast 15% skin 10% gastrointestinal 5% blood 5% brain 5% lymphoma	*Needs*: More sleep, social connections, leisure, and healthcare to address coping, reduce burden and fatigue; financial support; less traveling for treatment.	Difficulties traveling for treatment; geographic isolation; limited local access and specialized services; financial strain, limited care coordination and centralization.
Johnston et al. 2023 [40]	Australia	Investigate changes to employment for rural cancer patients and caregivers following cancer diagnosis and the impact that cancer and traveling for treatment has on their employment.	Mixed methods	211	48% 65 years or older 61% female	45% of rural sample from outer regional or remote areas, 55% from major city or inner regional area.	34% unspecified 18% breast 12% head and neck 12% prostate 13% skin 9% gynecological	*Problems*: Challenges keeping employment; social isolation and depression; difficulties balancing duties and making healthcare arrangements. *Needs*: Financial, employer, transportation, and social support.	Geographic remoteness, requiring travel; limited local services and treatment necessitating relocation; difficult to work remotely due to manual rural occupations; financial strain.
Johnston et al. 2022 [41]	Australia	Investigate sources of support sought by rural caregivers and factors associated with support‐seeking.	Cross‐sectional	244	62% female	All rural, outside major cities determined with ASGSRS 52% inner regional 42% outer regional 6% remote or very remote.	19% breast 14% skin 11% prostate 10% head and neck 9% gynecological 37% other or unknown	*Problems*: High caregiver burden, limited time. *Needs*: Psychosocial support for mental distress; caregiver support from general practitioner or online. *Other*: Male caregivers less likely to seek support.	Rural norms of self‐reliance and resiliency impeded mental health seeking and support; geographic isolation, limited healthcare access, financial barriers, time limited for responsibilities/work.
Kent et al. 2023 [20]	United States, southern region	Assess unmet social needs of family caregivers providing care to adults in rural settings.	Qualitative, interviews	24	Age mean = 54 years (range 20–67) 75% female 58% white 42% black	RUC codes 4+	83% stage IV solid tumor.	*Problems*: Social isolation, lack of staff connection. *Needs*: Economic (work, financial); physical (transportation, work issues, groceries, maintenance); interpersonal (friend and family support); service needs (transport, childcare, respite, legal, care coordination).	Geographic distance, travel burden, financial hardship intensified; few local specialists; lack of reliable transportation; social isolation due to distance from community when traveling; culture shock of travel. Gaps in support.
Lee et al. 2009 [42]	Australia	Identify common issues and explore the needs and experiences of people with mesothelioma and asbestos‐related lung cancer (M/ARLC), their carers, and service providers in the latrobe valley community.	Qualitative, interviews and media/employer reports	6	Demographics not provided.	Primarily rural area LaTrobe valley, but not assessed.	Mesothelioma and asbestos related lung cancer (M/ARLC).	*Problems*: High care burden; fatigue; emotional strain; social isolation. *Needs*: Education about disease.	Care delays and access gaps; travel burden for services; small pool of support; poor access to accurate information; stoicism led to delayed help‐seeking
Lewandowska et al. 2021 [43]	Poland	Describe the influence of cancer on patient and their caregiver. Analyze the problems and needs of patients in the early stages of primary treatment in all areas of life.	Cross‐sectional survey	800	Age mean = 57 years (SD = 7.8), range 31–85 years 88% women	36% rural (village, defined as 5000 or fewer residents) 64% city residents.	Solid tumor cancers only.	*Problems*: Fear, sadness, depression, and anxiety related to treatment; financial concerns; provider issues and insufficient information from providers; felt unprepared for patient problems across disease trajectory; *Needs*: Better access to healthcare services and resources; more reliable knowledge sources; financial needs	Lower information access
Perera et al. 2021 [44]	Australia	Identify strains experienced by urban and rural Australian informal cancer carers, as described by carers and providers, and to understand their views about a cancer carer‐specific online resource.	Qualitative, focus groups or interviews	20	14 female	Rural defined as areas outside major cities. 45% rural.	25% breast 15% blood 15% head and neck 10% skin 10% pancreas 5% liver 5% brain 5% lung 5% prostate 5% unknown	*Problems*: Psychosocial and financial strains; rural access difficult; social isolation; unprepared for tasks but then face multiple roles; challenging to balance family and home duties. *Needs*: Professional support.	Long travel times to care, limited access to support, limited online access; reluctance to seek help
Qian et al. 2023 [45]	United States	Illuminate the lived experience of resilience in rural‐dwelling north carolinian cancer caregivers at the intersection of cancer and the COVID‐19 pandemic.	Qualitative, interviews	24	75% female, 71% over age 50; age range 20–79. 42% black; 58% white	Rural zip code (RUC codes 4–9).	21% breast 17% hematologic 8% ovarian 8% colorectal 17% hematological 46% other Stage II: 8% Stage III: 8% Stage IV: 67%	*Problems*: Juggling responsibilities; financial issues; *Other*: Strong informal community support	Transportation issues (frequent 3–4‐h drives); cost of travel; rural roads susceptible to flooding or impassability; loss of work time; limited resources and services
Roydhouse et al., 2023 [46]	Australia	Understand the experience of people with multiple myeloma and myelodysplastic syndrome and their caregivers.	Qualitative, focus groups	16	Age mean = 71.5 years (46–84) 69% female	All rural, regional or remote in Tasmania, defined by Australian bureau of statistics.	72% multiple myeloma 28% myelodysplastic syndrome	*Needs*: Emotional and informational; feeling part of a care team; having a close relationship with the care recipient. *Other*: Social support received online; appreciated phone consultations from providers; church group support.	Geographic isolation; long travel distances; transportation/financial strain; limited local access
Schwartz et al. 2020 [47]	United States, North Carolina	Understand the barriers of rural older African American cancer patients and their caregivers, especially in adhering to treatment protocols.	Qualitative, interviews	8	75% female 100% African American	Specific rural counties, not further described.	Cancer type or stage not described.	*Problems*: Transportation; family duties; financial barriers; scheduling healthcare appointments. *Other*: Heavy reliance on family & community support	Limited public transportation; long wait times for transport; financial barriers for supplies; employment disruption; limited access to healthcare
Skalla et al. 2013 [48]	United States	Describe the multidimensional needs of diverse caregivers supporting cancer patients.	Cross‐sectional	172	Age mean = 54.9 years (SD = 11.5) 98% caucasian 84% female	Rural included but not described.	Cancer type or stage not described.	*Problems*: Fatigue, insomnia, weight gain, depression, anxiety; financial and transportation challenges. *Other*: Men have more unmet needs, but many did not want help.	Transportation barriers; financial strain; limited rural utilization in support services; employment disruption; limited support groups
Stiller et al. 2021 [49]	Australia	Describe the types and prevalence of supportive care needs of cancer caregivers in regional and remote Australia, both in terms of prevalence of needs and degree of unmet need and to compare unmet need according to the caregiver's age group, gender, geographical location, socio‐economic status, the length of time since diagnosis, and the cancer type of the patient.	Cross‐sectional	239	62% female 44% some college or more	Caring for cancer patients having to travel more than 50 km from home, staying 52.3% inner regional; 42.6% outer regional 5.1% remote/very remote.	18.4% breast 14.2% skin 11.7% prostate 10.5% head & neck 8.8% gynecological 6.7% colorectal 4.6% lung 5.4% lymphoma 2.1% brain 17.5% other or unknown	*Problems*: Physical health issues; mental health issues (anxiety, depression, anger, irritability, and nervousness); loneliness and isolation. *Needs*: Practical support; lodging during travel; treatment closer to home; financial support; respite care.	Travel challenges; distance to treatment; financial strain; limited local healthcare and psychological services
Tan et al. 2021 [50]	Australia	Synthesize the experiences of individuals and their caregivers in the face of uncertainty via ovarian cancer through thematic analysis.	Qualitative, open‐ended questions on survey	78	87% female 22% aged 30–39 years	29% of cancer patients rural or regional based upon postal codes, Australian Department of Health. Classification.	Ovarian cancer, 41% stage III	*Problems*: Fear of future; uncertainty; helplessness, disruption to life; isolation.	Lack of access to services and information; geographic isolation & treatment distance; limited specialist access and diagnosis delays; few peer/support groups or counseling services.
Tanriverdi et al. 2016 [51]	Turkey	Determine the personal, social, and economic burden and depression frequency among caregivers of cancer patients receiving chemotherapy.	Cross‐sectional	968	59% over age 50, range 18–73 years 56% female	58% rural, definition not described.	30% breast 20% colorectal 15% lung 48% metastatic disease	*Problems*: Depression; financial issues and employment loss; social isolation; lack of self‐care.	Limited oncology services; financial hardship due to loss of income and long distance travel; social isolation.
Tasman et al. 2023 [52]	United States, east Tennessee	Assess differences in patient psychological distress and caregiver strain between rural and non‐rural Appalachian breast‐cancer dyads.	Cross‐sectional	54	98% white	33% rural, self‐reported.	Breast cancer 54% stage I 33% stage II 11% stage III	*Problems*: Caregiver strain and anxiety.	Geographic isolation; travel burden; low access to oncology and mental health services.
Tolbert et al. 2018 [53]	United States	Discuss caregiving work up to and transition from initial care and beyond and explore needs of caregivers.	Qualitative, focus groups	19	74% female	53% rural, from rural community hospital study site.	Not provided.	*Problems*: Emotional and physical; relationship changes with patients. *Needs*: Involvement in healthcare processes with provider communications; written, comprehensible information about diagnosis and treatment decisions, and drug side effects; home support needs; self‐care help	Limited oncology & peer support services.
Ugalde et al. 2019 [54]	Australia	Understand rural caregiver experiences in supporting patients in accessing cancer treatment in metropolitan settings.	Qualitative, interviews	21	Age mean = 59.95 years (32–83) 76% female	100% rural, areas requiring 100+ km drive to access care.	29% hematological 24% gastrointestinal 14% bone/soft tissue 10% melanoma/skin 14% other (breast, lung, & gynecological)	*Problems*: Social estrangement; life adjustments; lack of care assistance, *Needs*: Culturally sensitive support; travel planning and accommodations.	Geographic isolation; farm and home maintenance upkeep; lack of confidence driving in city. Strong self‐reliance and stoicism norms; limited healthcare & psychosocial support access.
Williams et al., 2013 [55]	Canada	Describe the experience of family caregivers of persons with advanced cancer.	Qualitative, journal entries	23	Age mean = 59 years 100% female	Rural western Canada, self‐identified as rural.	Advanced cancer, not described.	*Problems*: Practical and emotional challenges; fatigue *Needs*: Ways to find hope and social support, such as faith group support; self‐care strategies, such as socializing and exercise.	Geographic isolation and long travel times; economic strain; fragmented rural services; limited professional and peer support; limited emergency access.
Xu et al. 2021 [56]	United States	Evaluate factors associated with caregiver mastery and caregiving burden among caregivers of older adults with advanced cancer, examine rural‐urban disparities in caregiver mastery and burden, and explore whether rural‐urban disparities in caregiver mastery and burden differed by caregiver education.	Cohort study	414	Age mean = 66.5 years 75% female 89% white	RUCA codes determined rurality. 15.5% were rural.	Advanced solid tumor or lymphoma.	*Problems*: Disrupted schedules; lack of rural mastery of skills; low caring confidence in rural areas; health problems. *Needs*: Social support, especially if less than high school education; employer support.	Lower education levels in rural areas; financial hardship, socioeconomic disadvantage, reduced social support; fewer community resources; workplace less flexible.

Eighteen included studies were from Australia, nine from the United States, and four from Canada, while the remainder came from other nations [47]. Most of the studies (*n* = 27) were published from 2015 to 2025, with eight from 2005 to 2014. Eighteen studies were qualitative designs; of those, 12 were from Australia, and five were from the U.S. Two of the qualitative studies from Australia utilized the same sample [38, 39], and there were three mixed‐methods studies from Australia [23, 26, 40].

Most studies reported primarily White samples; non‐U.S. studies generally included homogeneous populations or did not disclose participants' racial composition [42, 43, 55]. In contrast, the U.S.‐based studies had relatively more racial heterogeneity, with studies specific to rural American Indian and African‐American populations [36, 47].

The average caregiver age was not reported uniformly across studies, but when reported, the mean age ranged from 36.1 to 68 years. The lowest sample means were from U.S. and Canada studies with caregiver mean ages of 54–56.5 years. The highest mean ages were from Australian studies, with means of 66, 68, and 72, and one U.S. study had a mean age of 67 [33, 46, 56, 57]. Younger caregivers faced more fatigue and poorer psychosocial outcomes than older caregivers [27]. Caregivers were primarily female, and some studies included only female caregivers [37, 55, 58]. Male caregivers were identified as having unmet needs, but they did not always want or seek help even if available [41].

Several studies assessed the burden on caregivers of older (70+ years) cancer patients [31, 47, 56] or advanced cancer patients [28, 37, 55, 56]. Patients requiring assistance with activities of daily living or who had received more months of caregiver support presented a higher caregiver burden, either due to comorbidities or frailty, or disease severity [30]. However, these findings were nuanced as caregivers of recently diagnosed cancer patients also presented with significant levels of emotional distress [43], and caregiver stress was elevated even during shorter treatment periods [30].

The majority of the studies assessed caregivers of patients with any cancer, most commonly solid tumor cancers of the breast, colon, head and neck, and skin [23, 28, 29, 31, 33, 38, 39, 40, 41, 44, 45, 49, 51, 54]. However, some studies did not specify cancer type. Four studies included hematological cancer patient caregiver [27, 44, 45, 54], and nine did not provide details on patient cancers. Six samples included caregivers specific to patients with gastrointestinal, mesothelioma, and ovarian cancers [24, 26, 42, 50]. Few studies directly measured the unmet needs of cancer caregivers according to the patient's cancer, but one [49] found that breast cancer patient caregivers had the fewest unmet needs.

Of the 35 studies, 21 assessed rural‐only samples, whereas the remaining 14 included rural and urban individuals. Eleven studies did not explicitly define rurality, and other studies used a range of definitions, from rural to remote, or assessed self‐reported rurality. Eight studies used Australian standards of rurality, including the Australian Statistical Geographical Standards for rural and regional areas [23, 38, 39, 40, 41], the Accessibility Remoteness Index of Australia (ARIA) codes 3–5 [33] or 2–4 [52], the Australian Bureau of Statistics [46], and the Australian Department of Health standards [50]. Two studies in the United States used RU [20, 45] or RUC [56] codes to define rurality. Four studies used community size to determine rurality, with varying size limits of 10,000 residents, 30,000 residents, or no more than 1000 individuals per square mile [43]. Three studies considered rural as being at least 50 km from the nearest metropolitan center in two studies, and more than 300 km in another study. Two studies [52, 55] permitted participants to self‐identify as rural. Among studies that included rural and urban participants and provided a breakdown of rurality, the percentage of rural caregivers ranged from 15.5% to 73% [27, 56]. Several rural‐urban differences in unmet caregiver needs were identified, reflecting distinct challenges in the rural context. One study [56] found that urban caregivers in the U.S. reported higher self‐esteem and mastery of patient care skills, while rural caregivers reported more difficulties with finances, social support, and health. Rural caregivers in a southwestern American Indian sample faced more communication and cultural barriers, as well as physical barriers such as transportation, than urban caregivers [36]. One study identified rural challenges related to travel and a lack of support relative to urban areas [35]. Rural caregivers in Canada had higher caregiver burden scores and disability days than urban caregivers, as well as a higher likelihood of rural caregivers to utilize the emergency department over a physician for pain and symptom management [25]. Social isolation and access were common among rural ovarian cancer caregivers and patients in Australia [50].

### 3.1 Mental Distress

The most prevalent problem faced by informal cancer patient caregivers in rural areas was mental distress, defined as anxiety or depression, with 28 studies mentioning this problem, including 10 cross‐sectional studies [27, 28, 29, 30, 32, 43, 48, 49, 51, 52] and three mixed‐methods studies [23, 24, 26], with more qualitative studies providing evidence [20, 34, 35, 38, 39, 40, 41, 42, 44, 45, 46, 50, 53, 54, 55]. Caregiver anxiety reports varied in the samples [44, 48, 49], and were as high as 96% of caregivers reporting anxiety about patient needs in a cross‐sectional survey of Polish caregivers [43]. However, one study [32] identified that caregivers had no different levels of anxiety from the general population. Another noted that caregivers of mesothelioma patients indicated a high quality of life with no differences between rural and urban [29]. Several stated the need for more mental health services [24, 26, 41, 43, 44]. Depression was prevalent among both caregivers and patients [53], sometimes with the caregiver's depression worse than that of patients [23]. A qualitative study [44] supported this finding, noting that caregivers may experience greater distress as they frequently assume the burdens of their family member in addition to their own.

Caregivers had more depression and lower quality of life scores than the general population [28, 32]. Depression was identified in 50% of caregivers (rural and urban) in a cross‐sectional study, with 12% of the sample disclosing use of anti‐depressive pharmaceuticals [43], while a 58% rural sample of caregivers found that 64% disclosed depression [51].

Overall, both quantitative and qualitative studies identified significant emotional challenges among rural caregivers [24, 50, 55]. One study described a high emotional burden of caregiving, feeling that others did not understand what was happening, and the desire to avoid sympathy, along with the “grin and bear it” attitude [38]. Another study [30] identified that stress increased when patients had needs for activities of daily living. Several studies identified the negative impact of caregiving on the caregivers' mental health, acknowledging that rural caregivers were unlikely to seek help for their mental health issues, noting limited time and preference to focus on patient needs [34, 35, 59]. In one cross‐sectional study, self‐efficacy in dealing with difficult situations emerged as a mediating factor in improving rural caregivers' mental health [28].

### 3.2 Cultural Issues

Unique rural cultural issues affected caregivers, stemming from societal norms or perceived lack of understanding among healthcare providers. The majority of the 12 studies identifying cultural issues had qualitative designs [20, 34, 36, 38, 41, 42, 44, 54, 55], with limited quantitative evidence [23, 28, 30]. The rural norms of self‐sufficiency, stoicism, independence, and hesitancy to trust healthcare providers prevented rural caregivers, especially men, from seeking mental healthcare that they needed [33, 40, 42, 60]. Multiple caregivers felt a lack of empathy, culturally sensitive support, and awareness of the rural‐specific psychosocial stressors they faced when interacting with healthcare providers, and they wanted more rurally relevant information [23, 33, 34, 60]. As a result, caregivers were less likely to divulge information to healthcare providers and more likely to rely on family and social support [33]. When rural caregivers had diverse cultural backgrounds, their distress was increased [30, 36]. For instance, in one qualitative study, rural American Indians felt conflict when healthcare providers did not discuss stigmatized issues such as death and pain in a culturally appropriate manner [36].

### 3.3 Healthcare Provider Issues

Many rural caregivers had difficulties communicating or connecting with healthcare providers, particularly surrounding scheduling, accessing care or support services, and obtaining answers to patient care questions in 14 studies, mostly qualitative [20, 23, 24, 31, 33, 34, 35, 36, 37, 38, 43, 46, 47, 53]. One mixed‐methods study identified that rural caregivers felt disconnected from urban providers, experiencing poor service coordination and long wait times [23]. Other caregivers found care coordination difficult, often due to rural healthcare provider turnover, long wait times for social support services, and travel burden imposed by distance and multiple locations required for services, as two qualitative studies described [33, 34]. One qualitative study found that caregivers felt that their concerns mentioned to healthcare providers were put “in the drawer and that was it. They forgot about it.” [31].

In qualitative studies describing positive relationships between caregivers and healthcare providers, challenges were noted [37, 44, 53]. Caregivers valued communication but felt limited in their ability to interact with healthcare providers, perceiving conversations as patient‐centric and unidirectional. In cross‐sectional surveys of caregivers, there were high [47] information needs, with up to 79% of caregivers desiring more information and contact with healthcare providers [43, 50, 59].

### 3.4 Rural Access

In 29 of the 35 identified studies, caregivers reported limited rural access challenges in accessing services for themselves or the cancer patient they cared for. Seven studies, both qualitative and quantitative, specifically cited low rural access [23, 24, 31, 33, 42, 44, 47], with nearly half of caregivers identifying barriers to accessing services such as psychosocial support and difficulties scheduling specialist or general practitioner appointments, significant challenges in rural transportation increasing patient treatment non‐adherence, or limited cancer provider access. One qualitative study found that caregivers noted that travel accommodations and telehealth services could help them access healthcare [39]. Studies reported multiple concerns about access to healthcare providers due to rurality, including: longer appointment wait times, limited clinic hours, and delays to initial diagnoses, requiring caregivers to manage side effects independently or seek emergent treatment [24, 31, 33, 36, 38]. As one caregiver stated, “whenever we need somebody (locally), they're never here.” [31].

### 3.5 Lack of Care Skills

In nine studies [30, 35, 36, 37, 43, 44, 46, 53, 56], caregivers found that a lack of care skills was frustrating and desired more training; when they obtained confidence and mastery in patient care, they found it helpful in alleviating the perceived burden of providing care, such as assisting with basic activities, such as toileting and feeding, and administering oncolytic therapies and tube feedings to their family member, improving their emotions [30]. The caregivers wanted more information about patient diagnoses, symptom control across the cancer trajectory, diets, medication side effects, and complex needs such as ostomy tube management, to provide better patient care [37, 44, 53]. Multiple studies identified caregivers' desire to receive training from healthcare professionals or other caregivers, as they rapidly needed to upskill as the patient's disease progressed [35, 44]. In one study, 49% of caregivers reported being unprepared for patient problems [43]. A qualitative study described rural caregivers as “unprepared for the challenging burdens involved,” given the wide range of tasks they had to perform, from nursing duties to household management [44]. A cohort study reported a lack of mastery of care skills and the likelihood to seek help among rural caregivers, especially among less educated individuals [33, 56]. The study designs identifying skill needs were evenly split among designs that were cross‐sectional or mixed‐methods and qualitative.

### 3.6 Isolation and Loneliness

Multiple studies (22) identified social estrangement and isolation, low social support, caregiver loneliness, or feeling isolated in their roles and geographically separated from their communities [20, 23, 26, 27, 28, 31, 32, 33, 34, 36, 38, 39, 41, 42, 44, 49, 50, 51, 53, 54, 55, 61]. Few rural caregivers utilized available support services, although those participating in support groups found them helpful [34]. However, one study of 100 Canadian caregivers reported ample social support, although this was a small sample (*n* = 44) with a low response rate [25], and an Australian study (*n* = 16) identified online support and church groups as helpful in combating loneliness and isolation [46]. Approximately a third of the studies identifying isolation were cross‐sectional or mixed‐methods [26, 27, 28, 32, 49, 51], while the majority provided only qualitative evidence.

### 3.7 Fatigue or Physical Issues

Rural cancer caregivers had multiple physical health problems, including medical diagnoses, comorbidities, and general fatigue, and these were described evenly with quantitative and qualitative findings in 13 studies [23, 28, 30, 31, 32, 39, 42, 48, 49, 51, 53, 55, 56]. Studies comparing rural to urban caregivers found that physical issues were more prevalent among rural caregivers [44, 56]. Several qualitative studies noted fatigue or physical exhaustion as a theme [42, 53, 55]. In another mixed‐methods study, rural caregivers reported exhaustion [23].

Multiple studies (8) described rural caregivers as having trouble maintaining a healthy quality of life, as caregiving activities interfered with self‐care [28, 31, 32, 39, 51, 53, 56]. One study found that 28% of caregivers neglected their own health [49]. Another study found that younger caregivers were more likely to experience exhaustion and physical fatigue [30]. Few studies found mixed or positive health results among rural caregivers. One [39] study described that while rural caregivers were more physically active than urban caregivers, their overall health declined due to poor sleep and nutrition, and increased alcohol consumption.

### 3.8 Financial Issues

Overall, cancer caregiving led to a financial burden among rural caregivers, with one study identifying that 32% desired financial help [49]. This theme was supported by 17 qualitative, 9 quantitative, and one mixed‐methods studies. Rural‐dwelling caregivers of older adults reported economic issues [56], with up to 89% of a Polish sample of caregivers describing financial fears [43]. One U.S.‐based study of caregivers of rural African‐American patients described significant financial barriers to the cost of treatments, supplies, and medications, negatively affecting patient adherence to treatment plans [47].

Traveling difficulties led to increased financial issues or burden for rural caregivers in 26 identified studies, given the costs and lack of dependable transportation and lodging, and the stress of city driving [20, 31, 47, 60] Eleven studies specifically named transportation barriers or needs for support [20, 25, 33, 36, 39, 40, 45, 47, 48, 49, 54]. One study aptly described the rural caregiver situation, stating that “the distance and frequency of hospital visits compounded cancer‐related financial hardship, given additional costs of parking, gas, and lodging.” [45] A cross‐sectional study of rural U.S.‐based caregiver needs found that 30% of caregivers desired financial help with transportation [48], and another in rural Australia found that 77% of caregivers desired lodging closer to the hospital [49]. Travel increased caregivers' social isolation, prompting them to minimize travel [38, 39].

Financial and work issues were intertwined among caregivers, as caregiving made it challenging to maintain employment, thereby reducing household income [27, 33, 34, 45]. One study found that among employed rural caregivers, 37% were no longer working (62% stopped working, 38% retired), 31% reduced their hours, 28% took a leave of absence, and 11% changed their roles after a cancer diagnosis [40]. Those caregivers reporting a change in employment were significantly more likely to hold manual labor roles [40]. Sixteen studies described employment‐related needs of caregivers [20, 27, 29, 35, 36], suggesting that employer and childcare support would be beneficial [20, 29].

### 3.9 Disruption and Increased Struggles With Family/Home Duties

Several studies (20), mostly qualitative but including some quantitative, described difficulties that caregivers faced juggling previously shared household duties and childcare with limited family support at home [20, 24, 29, 30, 34, 35, 36, 38, 40, 41, 42, 44, 45, 47, 49, 50, 53, 54, 55]. Overall, rural caregivers had more disruption to their lives and schedules than urban caregivers, often needing to move or dramatically alter their home lives [24, 42, 45, 56]. Farm maintenance was identified as a challenge for rural caregivers, who faced daily obligations to care for livestock or manage property, already experiencing substantial stress and unpredictability due to weather and pricing, adding to their high caregiver burdens [34, 45].

### 3.10 Synthesis of Rural Contextual Factors Affecting Caregivers

Rural contextual factors were identified and extracted in Table 1. These results were then synthesized to identify how rurality affects caregiver demands and unmet needs (Table 2). Across the studies, structural factors intensified the burden of rural caregiving, especially for caregivers with long travel distances, geographic and social isolation, limited transportation options, and those who needed to relocate. Healthcare barriers were prominent, particularly in access to local specialized services and psychosocial services or support. These issues often expand caregiver responsibilities. Furthermore, other rurality‐related factors, such as less flexible employment and cultural norms of stoicism, affected the caregivers. These contributed to the underutilization of services. Overall, rural contextual factors amplified the primary problems and needs faced by the caregivers of cancer patients. To visually describe these findings, Figure 2 presents a model depicting how rural contextual factors increase caregiving demands and unmet needs, which then affect outcomes for caregivers.

**TABLE 2:** Cross‐study synthesis: Rural contextual factors in included studies.

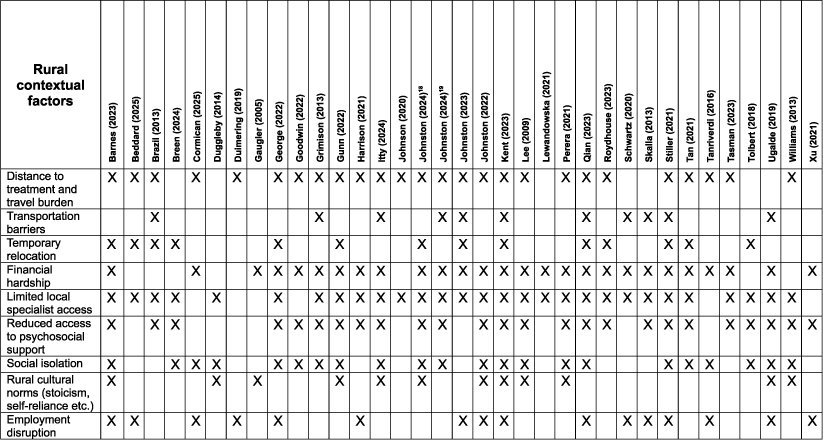

**FIGURE 2:**
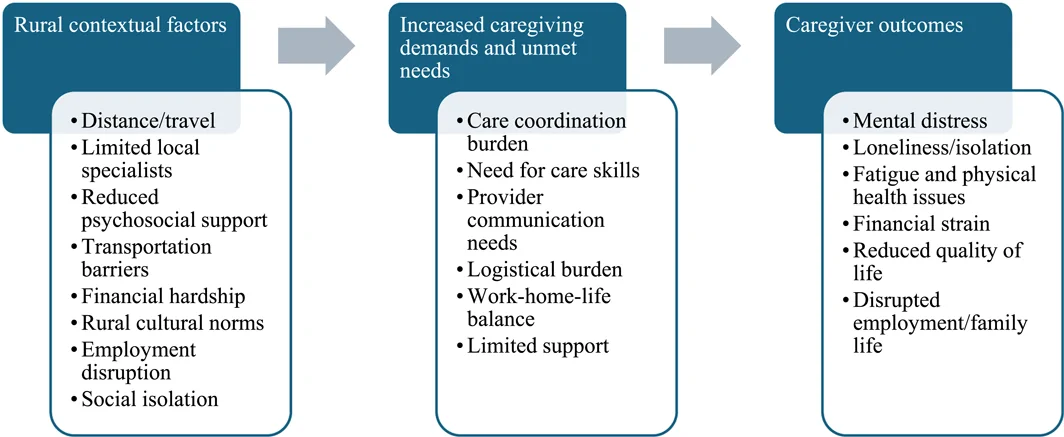
Rural contextual factors influencing caregiver needs and outcomes.

## 4 Discussion

Despite identifying several themes through primarily descriptive research in the 35 identified studies, the results of this scoping review demonstrate that research on caregivers of cancer patients in rural areas remains underdeveloped, especially given the rural burden of cancer caregiving. As the burden of cancer rises and more cancer treatments are delivered on an in‐home basis, the burden to caregivers is amplified as they oversee more medically complex tasks, and perform more patient monitoring and caregiving duties [62]. As the degree of care intensifies, due to either disease stage or age, decreasing the patient's ability to conduct activities of daily living, caregiver burden increases [30, 58]. Already, cancer caregivers average approximately 28.6 hours per week of care, with up to 42.5 hours per week during intensive disease stages, such as immediately after a surgery or during chemotherapy treatment [63]. By reviewing these studies, we have described the current state of the literature on rural caregivers, with the intent to improve the outcomes of the interconnected relationship between rural caregivers and cancer patients [62, 64].

Our review revealed that rural cancer caregivers have high levels of mental distress. They must navigate longer distances and have access to fewer resources, increasing their psychological burden [6]. The studies identified varied with the levels of caregiver depression, with anxiety more prevalent [62, 64, 65], and caregiver mental health outcomes varied according to patient acuity level. These findings are supported by evidence that rural populations in the U.S. have higher rates of depression and suicide compared to urban populations, due to reduced availability of mental healthcare [66]. Worse functional status and severe symptoms in patients lead to increased caregiver distress, which is exacerbated when they are located farther from clinical support [62]. Geographic distance could also explain why rural caregivers are less likely to seek help or social support for themselves, with limited time and multiple demands. However, rural caregivers may mask their struggles, consistent with other studies of rural groups, where help‐seeking for mental health is stigmatized or considered incongruent with the independent, stoic culture of rural areas [66]. While self‐efficacy mediates seeking mental health support [67], faith and religion also bolster rural caregivers' mental health, informing possible strategies to help rural caregivers [67].

Rural caregivers perceived healthcare providers as not always accommodating distance constraints, and multiple studies cited poor access, leaving rural caregivers to cope with urgent care needs independently or visit emergency departments. Rural caregivers had to navigate numerous barriers to get the cancer patient to appointments, etc. This highlights a potential need to address rural‐specific barriers within oncology practices, coordinate appointments, and ensure healthcare provider conversations specifically involve caregivers. These findings suggest that research could examine whether greater involvement of cancer navigators could help address caregiver needs for social support, self‐care, and care skills. Understanding the interaction between mental distress and caregiving burden, especially for rural cancer caregivers, is essential for designing effective interventions addressing their unique needs. However, a comprehensive approach that integrates the needs of rural cancer caregivers can inform strategies to address challenges. If these are tailored, such acceptable strategies can be incorporated into interventions that empower rural cancer caregivers.

Multiple studies cited financial, work, transportation, and time management as issues of rural cancer caregivers. Indeed, all caregivers face these when caring for a cancer patient, but rural caregivers face a more intense burden due to their isolated location. Higher travel expenses due to overnight stays, longer commutes, and increased driving overall, and increased chores around home or farm maintenance, caring for children, crops, and livestock must also be balanced. Caregiving often conflicts with other responsibilities, both paid and unpaid labor, and these can become major caregiver stressors, especially when there are rigid chore schedules, such as farm work to complete, which affect time allocation [68]. Additionally, many farms, especially small family‐operated ones, are put at risk when a family member is diagnosed with a condition incapacitating them, particularly if they hold expertise in livestock or equipment management. Outside of farming, the studies also identified that employment was challenging for rural caregivers to maintain, further exacerbating financial issues. These findings identify several areas that may warrant further evaluation, including respite care, travel accommodation support, detailed directions to appointments in unfamiliar cities, employer support, and clustered appointments and telehealth. Such strategies could potentially alleviate caregiver strains associated with employment, finances, and long‐distance travel, but their effectiveness for this population necessitates further study.

Rural caregiver needs are distinct and familiar across global populations, although some groups had specialized needs due to the rarity or severity of cancer diagnoses or stages. Cancer care was not explained in most studies, and there are nuances to caring for cancer patient receiving outpatient chemotherapy, oral oncolytics at home, and those with ostomies or other surgeries and sequelae. As care moves away from the clinical or institutional setting, caregivers endure additional burdens, and these complex duties and outcomes on both the caregiver and the cancer patient require exploration.

Many cancer caregiver needs are common across rural and urban contexts [14], including anxiety or depression, financial stress, and employment disruption, but these can be exacerbated in rural areas, also adding on the hurdles of longer distances, increased isolation, and reduced access to specialists or other psychosocial support. The rural context is essential to address in cancer patient caregivers, who face extra hurdles due to their locations, leading to unique cultural, social, and geographic factors, and disadvantages in care skills and provider access [6]. Increased rurality contributes to a greater burden in other caregiver studies [19]. Compared to overall research about caregivers of cancer patients, relatively little literature describes the current needs specific to rural caregivers of cancer patients in the U.S. The studies identified were overwhelmingly from Australia, using qualitative methods, and most lacked representative populations such as those found in the U.S., limiting conclusions. Still, these studies identified differences in the rural context that indicated needs for skill mastery and social support [56].

The findings from this scoping review demonstrate that additional research is needed, especially in the U.S., where 74% of the land surface and 14% of the population are rural, amid rural population aging and growth [69]. Although there are common themes among rural caregiver findings in Australia and the U.S., additional research is needed on rural U.S. cancer caregivers to identify their problems and unmet needs and develop customized interventions acceptable to these populations.

Several methodological limitations to the literature must also be considered when these findings are interpreted. Given that nearly all the evidence is from qualitative studies or cross‐sectional surveys, the conclusions about caregiver needs and experiences are limited due to prevalence and change over time. Further, while the predominant qualitative evidence provides rich experiential accounts, there is limited population‐level data. A lack of longitudinal data also limits understanding of how rural caregiver needs change over the trajectory of cancer, from diagnosis to survivorship, palliative or end‐of‐life care. Finally, there is limited intervention research in rural caregiver populations.

There were also limitations in the literature about sample representation and measurement, with predominantly White, female spousal caregivers, and underrepresented other groups. Rurality and caregiver outcomes were defined inconsistently across studies, with some geographic classifications used, and others relying on self‐described data. Even when official classifications were utilized, such as the ARIA, different thresholds were considered urban. Additionally, definitions of rural communities varied, ranging from caregivers living in communities with fewer than 5000 residents, to those with fewer than 30,000 residents, to other areas with a density of fewer than 1000 people per square mile. This heterogeneity in the definition of rural limits comparability across the evidence base.

Outcomes for caregivers included various measures of burden, distress, quality of life, and social support. Such differences in measurement makes it challenging to make cross‐study comparisons and prioritize future intervention goals. Future research requires the use of clearer rural definitions, more representative samples, longitudinal measurement designs, and standardized caregiver outcome measures.

### 4.1 Limitations

First, there may be studies not identified in these searches or in the gray literature. Further, access barriers such as transportation to rurally located cancer patients and their caregivers, as well as the care burden of cancer caregivers, may have impacted the studies' recruitment. We did not conduct a quality appraisal because this scoping review aimed to provide an overview of the topic and identify gaps. Further, while this review excluded bereaved caregivers, several of the studies focused on caregivers providing end‐of‐life or care to cancer patients who were older or with cancer types with poor prognoses, which could affect the outcomes, especially mental health outcomes, related to grief and depression. Several U.S. studies investigated niche rural populations of caregivers and cancer patients, such as American Indians, which are not generalizable to other rural populations. This scoping review, rather than serving as a comprehensive history of rural cancer caregiver needs, was limited to the last 20 years to ensure relevance to the current cancer caregiver landscape, particularly given changes in cancer treatment and survival, caregiving roles, and rural healthcare infrastructure, which can lead to survivorship and treatment bias, as more caregivers support patients living longer with cancer. Earlier studies may reflect more inpatient‐based care and fewer home‐based responsibilities, not the shift toward greater caregiver burden with the increase of outpatient chemotherapy, oral oncolytic therapies, and advancement of telehealth that is predominant in more recent studies. Thus, potential temporal bias may exist as unmet needs identified more recently may appear more prevalently as caregiving demands evolve, while earlier unmet needs, particularly those related to health disparities and rural access, could be underrepresented in the study selection.

### 4.2 Conclusions

This scoping review provides clarity and supplements evidence regarding common issues facing rural caregivers of cancer patients, including mental health, access, provider relations, and financial needs. It shares the shortcomings in the available research about this population. To improve outcomes for rural caregivers and indirectly benefit rural cancer patients, future research and interventions should explore the needs of rural caregivers across more U.S. locales to better elucidate cultural concerns across regions. Differences in needs among populations should be highlighted, especially by cancer type and sociodemographics. Further research will inform interventions addressing these groups' rural geographic and cultural distinctions. Finally, this review reveals the need for rurally competent care from healthcare providers interacting with cancer patient caregivers, as caregivers are well‐positioned to partner with providers to improve patients' well‐being.

### 4.3 Clinical Implications

This review's findings suggest several areas that clinicians and healthcare systems may consider when supporting rural cancer caregivers. These include assessing caregiver distress, skills, and physical health, explicitly involving caregivers in clinical communication and planning when appropriate, and tailoring education to the realities of rural caregiving, such as distance. Oncology nurses, social workers, and navigators may be well positioned to identify unmet needs of caregivers and connect them to support available. Strategies include providing anticipatory guidance, helping cluster appointments, telehealth, financial assistance, early referral to navigation, and connecting caregivers with respite or other home‐based services or referrals. These strategies could help address geographic and access‐based barriers facing rural caregivers and patients; however, their effectiveness requires evaluation. Importantly, caregiver support strategies should also be culturally competent and geographically responsive rather than strategies transferred directly from urban models.

## Author Contributions

**Jeanne M. Ward:** conceptualization, data curation, formal analysis, methodology, software, writing – original draft, review and editing. **Christa Torrisi:** formal analysis, software, validation, writing – review and editing, **Nicole Werner:** methodology, writing – reviewing and editing, **Victoria Champion:** conceptualization, writing – review and editing, funding acquisition.

## Funding

The National Cancer Institute of the National Institutes of Health supported this work. (T32CA117865: Champion, Mosher).

## Supporting information


**Table S1:** Search terms and strategies.

## Data Availability

The data that support the findings of this study are available from the corresponding author upon reasonable request.
