# Supplementary material for: Needs of Rural Caregivers of Patients With Cancer: A Scoping Review of Evidence From North America, Europe, and Australia
Source: Psychooncology. 2026 Oct 7;35(10):e70615. doi: 10.1002/pon.70615 (PMC13646025; doi:10.1002/pon.70615)
Supplement: Supplementary file 1 [file PON-35-e70615-s001.docx]

**Supplementary Table 1**

Search terms and strategies

| Database | Platform/search limits | Search terms | Results |
| --- | --- | --- | --- |
| PsycINFO  Search 1: 02/09/2025  Search 2: 09/04/2025 | EBSCOHost APA PsychInfo/2005-2-25, English-language, peer-reviewed | “caregiver” AND (“cancer” or “oncology”) AND (“caregiver burden” OR “Health Services Needs and Demands” OR problems or barriers OR needs) AND (“rural” or “remote”) | Search 1: 44  Search 2: 47 |
| CINAHL  Search 1: 02/09/2025  Search 2: 09/04/2025 | EBSCOHost CINAHL Complete/2005-2025, English-language, peer-reviewed | “caregiver” AND (“cancer” or “oncology”) AND (“caregiver burden” OR “Health Services Needs and Demands” OR problems or barriers OR needs) AND (“rural” or “remote”) | Search 1: 69  Search 2: 71 |
| EMBASE  Search 1: 02/09/2025  Search 2: 09/04/2025 | EMBASE (Elsevier)/2005-2025, articles, English-language | “caregiver” AND (“cancer” or “oncology”) AND (“caregiver burden” OR “Health Services Needs and Demands” OR problems or barriers OR needs) AND (“rural” or “remote”). Publication type: Article | Search 1: 181  Search 2: 186 |
| PubMed  Search 1: 02/09/2025  Search 2: 09/04/2025 | EBSCO/2005-2025, English-language, humans | “caregiver” [all fields] AND (“cancer” or “oncology” [MeSH]) AND (“caregiver burden” OR “Health Services Needs and Demands” [MeSH] OR problems or barriers OR needs) AND (“rural” or “remote”) | Search 1: 39  Search 2: 51 |
| Family & Society Studies Worldwide  Search 1: 02/09/2025  Search 2: 09/04/2025 | EBSCOHost Family & Society Studies Worldwide/2005-2025, English-language, peer-reviewed | “caregiver” AND (“cancer” or “oncology”) AND (“caregiver burden” OR “Health Services Needs and Demands” OR problems or barriers OR needs) AND (“rural” or “remote”) | Search 1: 44  Search 2: 46 |
| Google Scholar  Citation review: 09/03/2025 |  | All studies previously identified were searched for citations | Search 1: 19 articles identified for full-text review |
